# The Evaluation of Intrarenal Pressure Using a Novel Single-Use Flexible Ureteroscope with Live Intrarenal Pressure Monitoring—An Experimental Study in Porcine Models

**DOI:** 10.3390/life14091060

**Published:** 2024-08-24

**Authors:** Angelos Samaras, Vasileios Tatanis, Angelis Peteinaris, Mohammed Obaidat, Solon Faitatziadis, Athanasios Vagionis, Theodoros Spinos, Marina Mylonopoulou, Panagiotis Kallidonis, Evangelos Liatsikos

**Affiliations:** 1 Department of Urology, University Hospital of Patras, 26504 Rio, Greece; agelos.sam@gmail.com (A.S.); tatanisbas@gmail.com (V.T.); peteinarisaggelis@gmail.com (A.P.); kasious.klay@gmail.com (M.O.); solonasfait@gmail.com (S.F.); thanos_vagionis@hotmail.gr (A.V.); thspinos@otenet.gr (T.S.); marinamulonopoulou99@gmail.com (M.M.); pkallidonis@yahoo.com (P.K.); 2 Department of Urology, Medical University of Vienna, 1090 Vienna, Austria

**Keywords:** intrarenal pressure monitoring, ureteral access sheath, flexible ureteroscopy, in vivo, porcine models, ZebraScope

## Abstract

(1) Background: This study aims to evaluate how different irrigation settings and the use of ureteral access sheaths (UASs) of varying sizes impact intrarenal pressure (IRP) during flexible ureteroscopy (fURS) procedures in pigs. (2) Methods: This study utilized three anesthetized female pigs. A novel flexible ureteroscope with the ability to continuously record live intrarenal pressure was used to perform ureteroscopy in different settings. Ureteroscopy was performed without UAS and with the use of 11/13 and 12/14 UAS at the ureteropelvic junction. Two different irrigation methods were employed for each parameter: one using gravity flow and the other using manual pumping with a commercial pump. IRP was also recorded with the presence of a laser fiber or lithotripsy basket. (3) Results: The recorded mean IRP during flexible URS without UAS was 28.25 (±11.2) under gravity irrigation; 35.46 (±10.08) under manual pumping; 22.5 (±3.05) and 30.75 (±5.79) with a laser fiber under gravity irrigation and manual pumping, respectively; and 16.45 (±1.27) and 17.27 (±3.69) with a lithotripsy basket under gravity irrigation and manual pumping, respectively. With an 11/13 UAS, the mean IRP was 15.41 (±8.57) and 19.33 (±4.26) under gravity and manual pumping irrigation, respectively; 14.56 (±2.50) and 18.64 (±5.13) with a laser in each irrigation setting, respectively; and 13.10 (±3.39) and 13.86 (±4.63) with a lithotripsy basket, respectively. With a 12/14 UAS, the mean IRP was 7.64 (±3.08) and 9.25 (±1.42) under gravity and manual pumping irrigation, respectively; 9.50 (±6.04) and 10.28 (3.46), respectively, in each setting when the laser fiber was used; and 5.32 (±1.57) and 6.26 (±1.79), respectively, when the lithotripsy basket was inserted. (4) Conclusion: Novel flexible ureteroscopes with integrated pressure sensors are both a feasible and reliable tool during fURS, giving the surgeon the ability to live-track the IRP. The results of the IRP measurements with and without UAS are in accordance with the current literature and exhibit a consistent pattern with previous studies.

## 1. Introduction

The endoscopic approach for the management of either ureteral or renal stones is the method of choice nowadays and is supported by international guidelines [1,2]. Innovative endoscopy tools have increased the effectiveness and safety of ureteroscopy and have established it as a routine treatment worldwide [3]. Although rigid and semi-rigid ureteroscopes seem to have reached the peak of their development, the same is not true for flexible ureteroscopes, whose evolution is continuous [3]. The ongoing improvement in flexible ureteroscopes has contributed to both the diagnosis and treatment of diseases of the upper urinary tract, especially urinary tract stones, independently to some extent by their size, location, and number [4,5]. The reduction in the diameter of the tools and the improvement in the optical quality are some of the most important developments [4]. Another critical innovation is the suctioning ureteral access sheath (UAS), which has been shown to maintain low renal pelvic pressure, while the safety and efficacy of lithotripsy is improved [6,7]. The level of visibility and durability has also improved, while efforts to miniaturize tools continue [3]. In order to achieve optimal visibility during an endoscopic procedure, the irrigation flow and irrigation pressure should be increased; however, this procedure is associated with the risk of complications [5,8,9]. Although the limit of intrarenal pressure (IRP) at which complications are observed is not sufficiently clarified, it is recommended not to exceed 40 cm H_2_O [5,8,9,10]. Despite the use of suction, ureteroscopy with a flexible ureteroscope can lead to a significant increase in IRP due to excessive fluid flow, which in turn may lead to complications, such as pyelovenous and pyelolymphatic reflux, renal tissue damage, systemic inflammatory response syndrome (SIRS), and sepsis [9,10,11].

So far, no tool has offered the ability of live tracking of the measurement of the IRP during ureteroscopies. Τhe use of new flexible ureteroscopes, which have an integrated intrarenal pressure sensor, offers new perspectives. This study aims to evaluate the results from the use of a novel flexible ureteroscope (ZebraScope^®^, Happiness Works, Bengbu, China), which has the ability to continuously record live intrarenal pressure during fURS, while also warning the operator in case the pressure limit is exceeded.

## 2. Materials and Methods

### 2.1. Study Design and Experimental Setting

Following approval from the relevant Veterinary State Services, an experimental setup was established to measure live intrarenal pressure during flexible ureteroscopy. The procedure utilized a novel flexible ureteroscope capable of continuously recording real-time intrarenal pressure. Various sizes of ureteral access sheaths (UASs) and different irrigation methods were tested in three female pigs. The median weight of the pigs was 30 kg (range, 28–32 kg). The pigs were crossbreeds from Danish Landrace and American Duroc species and the mean age was 12 weeks old (range, 10–13 weeks). The number of animals was not determined through a statistical analysis. Instead, it was considered that three pigs, which allowed for a total of six procedures, would provide sufficient data for reliable results, given the limited availability of additional animals.

**Figure 1:**
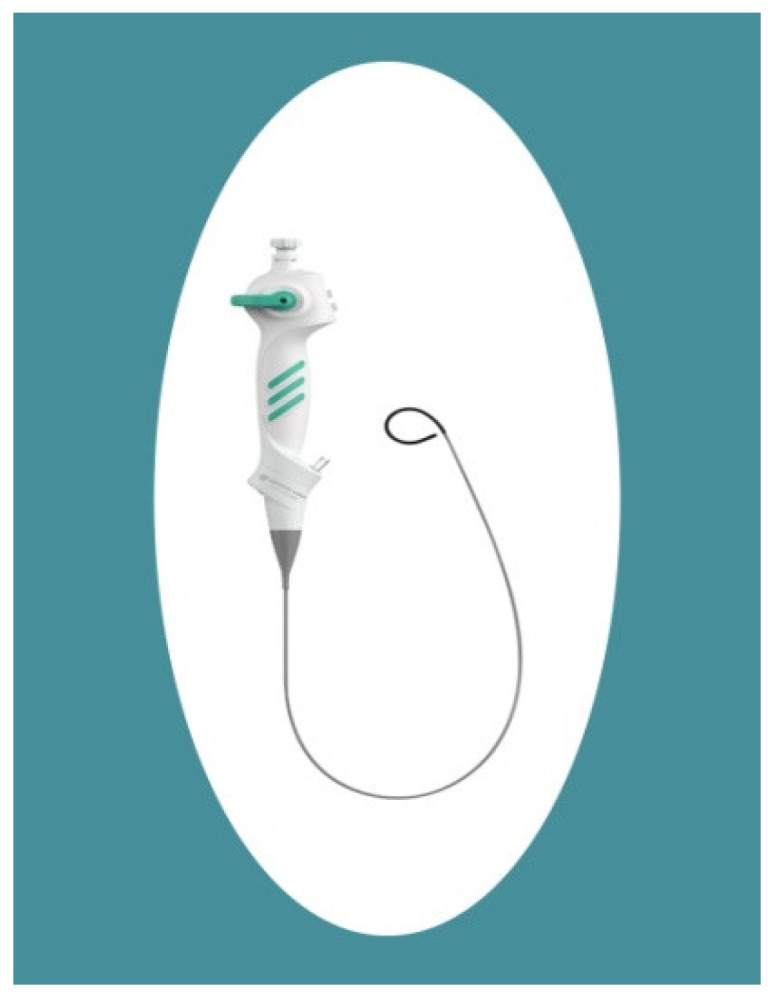
ZebraScope ureteroscope.

ZebraScope^®^ (Happiness Works, Anhui, China) is a novel single-use digital flexible ureteroscope (Figure 1), which has an integrated pressure monitor, offering the ability to sense intrarenal pressure in real time. IRP data are displayed per second on the operating room monitor, enabling immediate awareness of the pressure within the renal system. It has an 8.6Fr outer diameter, a 670 mm length of insertion, and a 275° bending angle. 

### 2.2. Preparation of the Pigs for the Experiment

The husbandry conditions were in accordance with veterinary guidelines. The feed was based on corn (50%), which contained wheat bran, barley, soybean meal, and a mix of vitamins and trace elements. Food was withheld for 12 h prior to the experiment. Each pig was placed in a prone position on the operating table, and an intravenous line was established in an ear vein. Anesthesia was initiated using a combination of Ketamine, Xylazine, and Atropine Sulfate. Following this, an intubation tube was inserted and connected to a ventilator. The anesthesia was maintained with a continuous intravenous infusion of 5% propofol throughout the procedure.

### 2.3. Operating Room Setup and Technique (Figure 2)

The pig was positioned supine for the procedure. Cystoscopy was performed to locate the ureteral orifice. A 0.035-inch hydrophilic guidewire (HiWire™ Nitinol Core Wire Guide, COOK Medical, Cook Ireland Ltd., Limerick, Ireland) was advanced to the kidney, and a 6F open-end ureteral catheter (COOK Medical, Cook Ireland Ltd., Limerick, Ireland) was placed over the guidewire, with the catheter tip positioned in either the upper calyx or the renal pelvis. A contrast injection was then administered to outline the pelvicalyceal system (PCS). To differentiate between the kidneys, blue and red markers were attached to the ureteral catheters for the right and left kidneys, respectively. Both catheters were secured to the external genitalia using 2/0 Silk sutures to prevent displacement.

**Figure 2:**
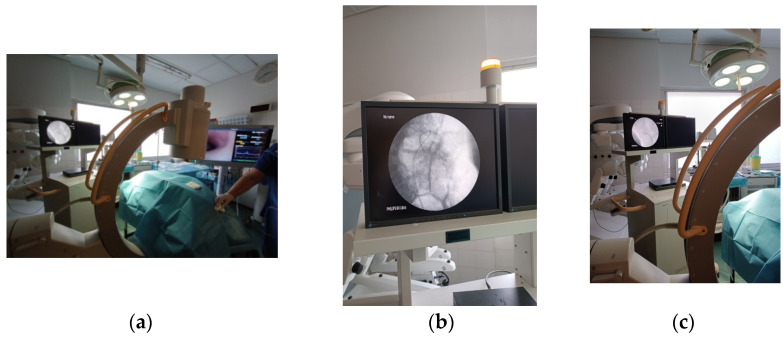
Operating room setup.

### 2.4. Measurement of the IRP

**Figure 3:**
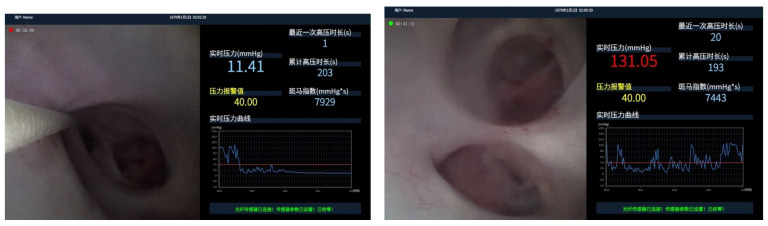
Example of user interface. Real-time IRP value over 11.41 and 131.05. Preset IRP Threshold over 40.00. Real-time IRP Curve over the curve. Recent High IRP duration (seconds) over 1 and 20. Accumulate High IRP duration over 203 and 193. Zebra index over 7929 and 7443.

Two irrigation settings were examined: continuous irrigation with a 3L bag positioned 1 m above the operating table (gravity irrigation) and manual pumping irrigation. The IRP was continuously monitored during flexible ureteroscopy, both without a UAS and with UAS sizes 11/13 and 12/14 (Flexor^®^ Ureteral Access Sheath with AQ^®^ Hydrophilic Coating, COOK Medical, Cook Ireland Ltd., Limerick, Ireland) placed just below the uretero-pelvic junction. The IRP was also recorded during the use of two more working channel accessories (a lithotripsy basket as well as a laser fiber) in the above-mentioned settings. Each measurement was recorded over a 1 min period per setting (Figure 3). 

### 2.5. Statistical Analysis

The pressure outcomes were collected and presented as the mean value and standard deviation. The statistical analysis was performed using the ANOVA one-way test. Significance was set at a *p*-value of <0.05. The statistical analysis was performed utilizing the Statistical Package for the Social Sciences (SPSS) software package version 25.0 (IBM Corp., Armonk, NY, USA).

## 3. Results

IRP mean values as well as the statistical difference (SD) are shown for the 11/13 and 12/14 UAS and without any UAS (Table 1). 

IRP was the highest without the use of UAS. Under gravity irrigation, the recorded mean IRP was 28.25 (±11.2) mmHg. On the other hand, under manual pumping irrigation, the recorded mean IRP was 35.46 (±10.08) mmHg. When a laser fiber was inserted, the mean IRP was 22.5 (±3.05) mmHg under gravity irrigation. When changing to manual pumping irrigation, the recorded mean IRP was calculated at 30.75 (±5.79) mmHg. Furthermore, a lithotripsy basket was inserted and the two irrigation settings were performed. Using gravity irrigation, the mean IRP was estimated to be 16.45 (±1.27) mmHg, while under manual pumping irrigation, the recorded mean IRP was 17.27 (±3.69) mmHg.

The use of UAS proved to reduce the IRP. Firstly, the 11/13 UAS was examined. Under normal irrigation and manual pumping irrigation, the recorded mean IRPs were 15.41 (±8.57) mmHg and 19.33 (±4.26) mmHg, respectively. When the laser fiber was inserted, the recorded mean IRPs were 14.56 (±2.50) mmHg and 18.64 (±5.13) mmHg, respectively. When the lithotripsy basket was inserted and the two irrigation settings were performed, the recorded mean IRPs were 13.10 (±3.39) mmHg and 13.86 (±4.63) mmHg, respectively. 

Lastly, the 12/14 UAS was evaluated, which further reduced the mean IRPs. The recorded IRPs were 7.64 (±3.08) mmHg and 9.25 (±1.42) mmHg under normal irrigation and manual pumping irrigation, respectively. IRP measurements of 9.50 (±6.04) mmHg and 10.28 (3.46) mmHg were recorded in each setting when the laser fiber was used, while they were 5.32 (±1.57) mmHg and 6.26 (±1.79) mmHg, respectively, when the lithotripsy basket was inserted. 

The statistical analysis between the different UAS for each setting proved that the use of a wider-access sheath is associated with statistically significantly reduced IRPs. The *p*-value was <0.001 for every setting comparison (Table 1).

## 4. Discussion

Ureteral access sheaths are considered efficient tools for the decrease in IRP, by diverting the irrigation fluid stream externally, while significantly higher IRPs could occur during URS without the use of UAS [5,8,11]. Although the use of UAS is still debated, the main advantages of using UAS are the facilitation of repeated entrances in the ureter and the pelvicalyceal system, the protection of the ureteroscope, the protection of the ureter when extracting stone fragments, and the decrease in IRP [10,11,12,13,14]. However, complications may also occur, especially with the use of large-diameter UAS, such as ureteral injury and ischemia of the ureteral mucosa, due to the effect on the ureteral blood flow [10,12,13]. In our study, we assessed the effect of irrigation settings and the use of UAS of different sizes on the IRP during flexible ureteroscopy in porcine models. To our knowledge, the literature on the assessment of the IRP using a flexible ureteroscope with an integrated pressure sensor in live anesthetized pigs and comparing the use of UAS, as well as different UAS diameters and irrigation settings, is restricted. 

In our study, the highest IRPs were recorded in all irrigation settings without the use of UAS, most probably due to the insufficient diversion of the irrigation fluid stream externally. In all cases that a UAS was used, IRP remained below the limit of 30 cm H_2_O. Lower IRP values were recorded in all irrigation settings when 12/14 UAS was used in comparison with the use of 11/13 UAS, although in both cases the mean IRP values were still lower than the IRP values without UAS. Among the irrigation methods that were used, IRPs were lower in all settings when gravity irrigation was used in comparison with manual pumping, possibly because the diameter of a normal ureter cannot accommodate all the irrigation fluid and the pressure is transmitted in the pyelocalyceal system. Moreover, the use of either a laser fiber or lithotripsy basket proved to decrease the IRP in comparison with irrigation flow without a tool in the ureteroscope, possibly because of the occupation of the working channel. Throughout the procedures, no complications, such as perforation or hemorrhage, were encountered. Visibility was sufficient in all examined settings, with the clearest view obtained through manual pumping. Additionally, we observed that lowering the IRP during the fURS might reduce visibility, but it is unlikely to reach a degree that would hinder experienced surgeons from carrying out the procedure effectively. However, as visibility was not the main focus of our study, additional research is necessary to explore this aspect more comprehensively. 

The results of our study agree with a previous study of our clinic, in which the effect of irrigation settings and the size of UAS on the maximal intra-pelvic pressure (IPPmax) during ureteroscopy in pigs was assessed, by measuring the IPP through the creation of percutaneous access and placement of a nephrostomy tube to which a urodynamic pressure measuring device was attached [13]. In this study, Noureldin YA et al. concluded that the wider the diameter of the UAS, the lower the IPPmax, even under forced irrigation during fURS. Furthermore, manual pumping significantly increased the IPPmax in comparison with gravity flow irrigation. Thus, it is demonstrated by both studies that performing fURS using UAS can significantly reduce the IRP, which as a result decreases the chances of complications associated with high IRP. 

Until now, IRP during fURS was not monitored, because no tool had this capability. Only a few methods have been tested so far, in order to measure IRP during fURS, although the perfect system for daily practice has not been developed yet [15]. A ureteral catheter connected to a transducer, although easily implementable, leaves no space inside the ureter [15]. Sensor wires have also been proposed, but they are currently not approved for endourological procedures [15]. UASs, which integrate both suction/irrigation and IRP measurement, have been evaluated, having an alarm when the IRP is increased over the limit [15]. Bai J et al. used a fiber-optic pressure sensor device to monitor IRP during fURS in six porcine models [16]. It was concluded that the IRP does not exceed the 30 mmHg threshold when an 11/13 UAS is used, although the IRP is increased by manual or syringe pumping, as well as gravity irrigation >1,1 m in height. Similarly, in our study, in all cases that a UAS was used, mean IRP remained below the limit of 30 cm H_2_O. In our study, a novel flexible ureteroscope with an integrated intrarenal pressure sensor was used. ZebraScope^®^ offers the ability to continuously record live IRP by using a pressure sensor at the tip of the ureteroscope. This has the advantage of monitoring the intrarenal pressure second by second and enables the immediate intraoperative reaction in case of an excessive intrarenal pressure increase. Searching the literature, only one study was found in which those novel ureteroscopes were used. Chew BH et al. examined a similar ureteroscope in one porcine model [17]. The IRP was measured with and without a UAS in a variety of sizes and irrigation methods. The results were comparable to our study. The highest pressures were recorded during fURS without UAS, followed by the 11/13 and the 12 and 13/15 UAS. 

Previous studies in the past have tried to analyze IRP and the effect of UAS on it. In their systematic review, De Coninck V et al. reported that because of increased irrigation outflow during fURS with UAS, IRP remains below 30 cm H_2_O, and the irrigation pressure transmitted to the pyelocalyceal system is decreased by 57–75% [10]. They also suggested that the more the UAS size decreases, the higher the IRP for the same flexible ureteroscope. However, using a small-sized flexible ureteroscope could also prevent high IRP during fURS with small-sized UAS. Similarly, in our study, the mean IRP when an 11/13 UAS was used was always higher in comparison to the use of 12/14 UAS in each setting. In another systematic review, Breda A. et al. concluded that among other advantages, UAS facilitates the decrease in IRP, while protecting both the ureter and the ureteroscope and expediting stone extraction [14]. Rehman J. et al., evaluating the impact of UAS in IRP during fURS in cadaveric kidneys, concluded that using either a 10/12, a 12/14, or a 14/16 Fr UAS with pressurized irrigation improves visibility, while IRP is maintained below 40 cm H_2_O, due to the rapid outflow through the UAS [18]. Moreover, they reported an increase of up to 80% in irrigation flow when using UAS, in contrast with fURS without UAS. Indeed, in our study, the mean IRP never exceeded 40 cm H_2_O in any setting when a UAS was used under manual pumping irrigation. Pauchard F et al. reported in their review that UAS reduces IRP when a sheath of adequate diameter with the equipment is used [19]. IRP is also decreased when the irrigation flow is low and the working channel is occupied by a laser or basket. De Coninck V et al. also confirmed in their systematic review that the use of UAS is associated with lower IRP, but according to the study, UAS should not be used routinely during RIRS, but rather be reserved for cases with difficult ureteral access, low visibility during the procedure, and lithiasis treatment in patients with a high risk of infectious complications [20]. 

According to our knowledge, the current study is one of the first to assess the IRP in live anesthetized pigs, comparing the use of UAS, as well as different UAS diameters and irrigation settings, although it has some limitations, such as the small number of pigs, the deterioration of the pig kidneys after repeated tries, and the variability in the force applied during the manual pumping. However, pigs offer a similar urologic anatomy and physiology to humans and the IRP is affected by similar physiological factors, such as pyelorenal backflow. Therefore, normal physiologic conditions are ensured and any bias due to the use of porcine models and ex vivo human cadavers is avoided. 

## 5. Conclusions

Novel flexible ureteroscopes with integrated pressure sensors are both a feasible and reliable tool during fURS, giving the operator the ability of live tracking the IRP. The results of the IRP measurements with and without UAS are in accordance with the current literature and exhibit a consistent pattern with previous studies. 

## Figures and Tables

**Table 1:** Intrarenal Pressures under different settings (The mean values ± SD are presented).

Type of Setting	Without UAS	11/13 UAS	12/14 UAS	*p*-Value
Gravity irrigation	28.25 ± 11.2 mmHg	15.41 ± 8.57 mmHg	7.64 ± 3.08 mmHg	*p* < 0.001
Manual pump irrigation	35.46 ± 10.08 mmHg	19.33 ± 4.26 mmHg	9.25 ± 1.42 mmHg	*p* < 0.001
Laser fiber + gravity irrigation	22.5 ± 3.05 mmHg	14.56 ± 2.5 mmHg	9.50 ± 6.04 mmHg	*p* < 0.001
Laser fiber + manual pump irrigation	30.75 ± 5.79 mmHg	18.64 ± 5.13 mmHg	10.28 ± 3.46 mmHg	*p* < 0.001
Basket + gravity irrigation	16.45 ± 1.27 mmHg	13.10 ± 3.39 mmHg	5.32 ± 1.57 mmHg	*p* < 0.001
Basket + manual pump irrigation	17.27 ± 3.69 mmHg	13.86 ± 4.63 mmHg	6.26 ± 1.79 mmHg	*p* < 0.001

## Data Availability

The data presented in this study are available on request from the corresponding author.
